# MRI characteristics of ovarian metastasis: differentiation from stomach and colorectal cancer

**DOI:** 10.1007/s11604-024-01700-6

**Published:** 2024-11-14

**Authors:** Yukiko Takai, Hiroki Kato, Masaya Kawaguchi, Kazuhiro Kobayashi, Kyoko Kikuno, Tatsuro Furui, Masanori Isobe, Yoshifumi Noda, Fuminori Hyodo, Masayuki Matsuo

**Affiliations:** 1 Department of Radiology, Gifu University, 1-1 Yanagido, Gifu, 501-1194 Japan; 2 Department of Pathology, Gifu University, Gifu, Japan; 3 Department of Obstetrics and Gynecology, Gifu University, Gifu, Japan; 4 Department of Frontier Science for Imaging, Gifu University, Gifu, Japan; 5 Department of Pharmacology, School of Medicine, Gifu University, Gifu, Japan; 6 Center for One Medicine Innovative Translational Research (COMIT), Institute for Advanced Study, Gifu University, Gifu, Japan

**Keywords:** Ovarian metastasis, Stomach cancer, Colorectal cancer, Black scrunchie sign, MRI

## Abstract

**Purpose:**

To evaluate the efficacy of MRI findings for differentiating between ovarian metastasis from stomach cancer (OMSC) and colorectal cancer (OMCC).

**Methods:**

Twenty-six patients with histopathologically proven ovarian metastasis (*n* = 8 with 12 OMSCs and *n* = 18 with 25 OMCCs) were enrolled in the study. All patients had undergone pelvic MRI before surgery. We retrospectively reviewed MRI findings and compared them between the two pathologies. The black scrunchie sign was defined as a thick (> 5 mm) and lobulated hypointense rim (> 180°) with central hyperintense areas on T2-weighted images.

**Results:**

Predominantly solid lesions (100% vs. 20%, *p* < 0.01), black scrunchie sign (33% vs. 0%, *p* < 0.01), and flow void (67% vs. 20%, *p* < 0.01) were frequently observed in OMSCs than in OMCCs. The signal intensity ratio of solid components on T2-weighted images (3.30 ± 0.70 vs. 2.52 ± 0.77, *p* < 0.01) and gadolinium-enhanced T1-weighted images (2.21 ± 0.57 vs. 1.43 ± 0.32, *p* < 0.01) were significantly higher in OMSCs than in OMCCs. Furthermore, hyperintense areas within cystic components on T1-weighted images (71% vs. 18%, *p* < 0.01) and stained-glass appearance (44% vs. 0%, *p* < 0.01) were frequently observed in OMCCs than in OMSCs.

**Conclusion:**

The black scrunchie sign was only observed in OMSCs. OMSCs always exhibited predominantly solid lesions and had higher signal intensity of solid components on T2- and gadolinium-enhanced T1-weighted images. OMCCs usually presented as cystic lesions, usually accompanied by hyperintense areas within the cystic components on T1-weighted images.

## Introduction

Metastatic ovarian tumors are relatively common and account for 5%–30% of malignant ovarian tumors. Compared with primary ovarian cancers, they are more prevalent in premenopausal women and tend to be shown either bilateral or unilateral lesions. The colorectum and stomach are the most common nongynecologic primary tumor sites, followed by the breast, appendix, uterus, pancreas, and bile duct. The detection of ovarian metastasis precedes the diagnosis of the primary tumor in up to 40% of cases, especially in patients with colon and stomach cancer [1].

Krukenberg tumors are ovarian metastases from primary mucin-secreting signet-ring cell carcinomas. They account for approximately 30–40% of all ovarian metastases. The stomach is the most common primary tumor site for Krukenberg tumors, followed by the colorectal, breast, biliary tract and gallbladder, and by other sites (small intestine, appendix, pancreas, uterus, urinary bladder, renal pelvis) [2]. Retrograde lymphatic spread is believed to be the most likely route for metastasis in Krukenberg tumors [2].

The standard treatment for patients with ovarian metastasis has not yet been established; however, metastasectomy may be associated with longer survival benefits in patients with OMSC and ovarian metastasis from colorectal cancer (OMCC) [3, 4]. Ovarian metastasis is characterized by poor prognosis; the prognosis is considerably different depending on the primary sites. Specifically, the median overall survival of patients with ovarian metastasis was 11 months for stomach cancer and 21.5 months for colorectal cancer [5]. Therefore, accurate differentiation between OMSC and OMCC is important for predicting patient prognosis. The differences in CT findings between OMSC and OMCC [6], those in MRI findings between metastatic ovarian tumors and primary ovarian cancers [7–9], and the characteristic imaging findings of ovarian metastasis [10, 11] have been reported. However, to the best of our knowledge, no previous studies have examined the differences in MRI findings between OMSC and OMCC. Therefore, this study aimed to evaluate the efficacy of MRI findings for differentiating between OMSC and OMCC.

## Methods

### Patients

The study was approved by the human research committee of our institutional review board and complied with the guidelines of the Health Insurance Portability and Accountability Act. The requirement for informed consent was waived due to the retrospective nature of this study. Thirty-five patients with histopathologically proven stomach or colorectal cancer and clinically suspected of having ovarian metastasis were identified from the electronic medical record database of our hospital between April 2006 and December 2023. Among them, we excluded seven and two patients who had not undergo metastasectomy and pelvic MRI before surgery, respectively. Consequently, this study included 26 patients with ovarian metastasis (8 with stomach cancer and 18 with colorectal cancer) who had undergone metastasectomy and preoperative pelvic MRI. The primary sites of colon cancer were the sigmoid colon (*n* = 7), rectum (*n* = 6), transverse colon (*n* = 2), cecum (*n* = 2), and descending colon (*n* = 1). Age and serum tumor marker levels, including serum carcinoembryonic antigen (CEA), cancer antigen 125 (CA125), and carbohydrate antigen 19-9 (CA19-9) were recorded. CEA was examined in 25 of 26 patients, CA125 in 22 of 26 patients, and CA19-9 in all patients.

### Imaging technique

MRI was performed for all patients using 1.5 Tesla MRI scanners (Intera Achieva 1.5 T Pulsar, Philips Healthcare, Best, the Netherlands or Inginea Prodiva 1.5 T CS, Philips Healthcare, Best, the Netherlands) or a 3.0 Tesla MRI scanner (Intera Achieva 3.0 T Quasar Dual, Philips Healthcare, Best, The Netherlands). All images were obtained at a section thickness of 4–8 mm with an intersection gap of 1–2 mm. Axial, coronal, and sagittal non-fat-suppressed T2-weighted images (TR/TE, 3000–5923/90–100 ms) and axial non-fat-suppressed T1-weighted images (TR/TE, 502–728/10–15 ms) were obtained in all patients. Axial short-tau inversion recovery single-shot spin-echo echo-planar diffusion-weighted images (TR/TE, 4000–5000/55–73 ms) with b values of 0 and 1000 s/mm were obtained in 22 patients (seven with stomach cancer and 15 with colorectal cancer). Axial and sagittal fat-suppressed contrast-enhanced T1-weighted images (TR/TE, 548–769/10–17 ms) were obtained in 14 patients (six with stomach cancer and eight with colorectal cancer) after the intravenous injection of 0.1 mmol/kg of gadopentetate dimeglumine (Magnevist; Bayer HealthCare, Leverkusen, Germany) or gadobutrol (Gadavist; Bayer HealthCare, Leverkusen, Germany).

### Imaging assessment

Two radiologists (Radiologists 1 and 2) with post-training experience in gynecological imaging of 24 and 10 years, respectively, reviewed all MRI images individually and randomly. They were unaware of any clinical information or pathological diagnosis. Any disagreement between the two reviewers was resolved via discussion until a consensus was reached.

**Fig. 1:**
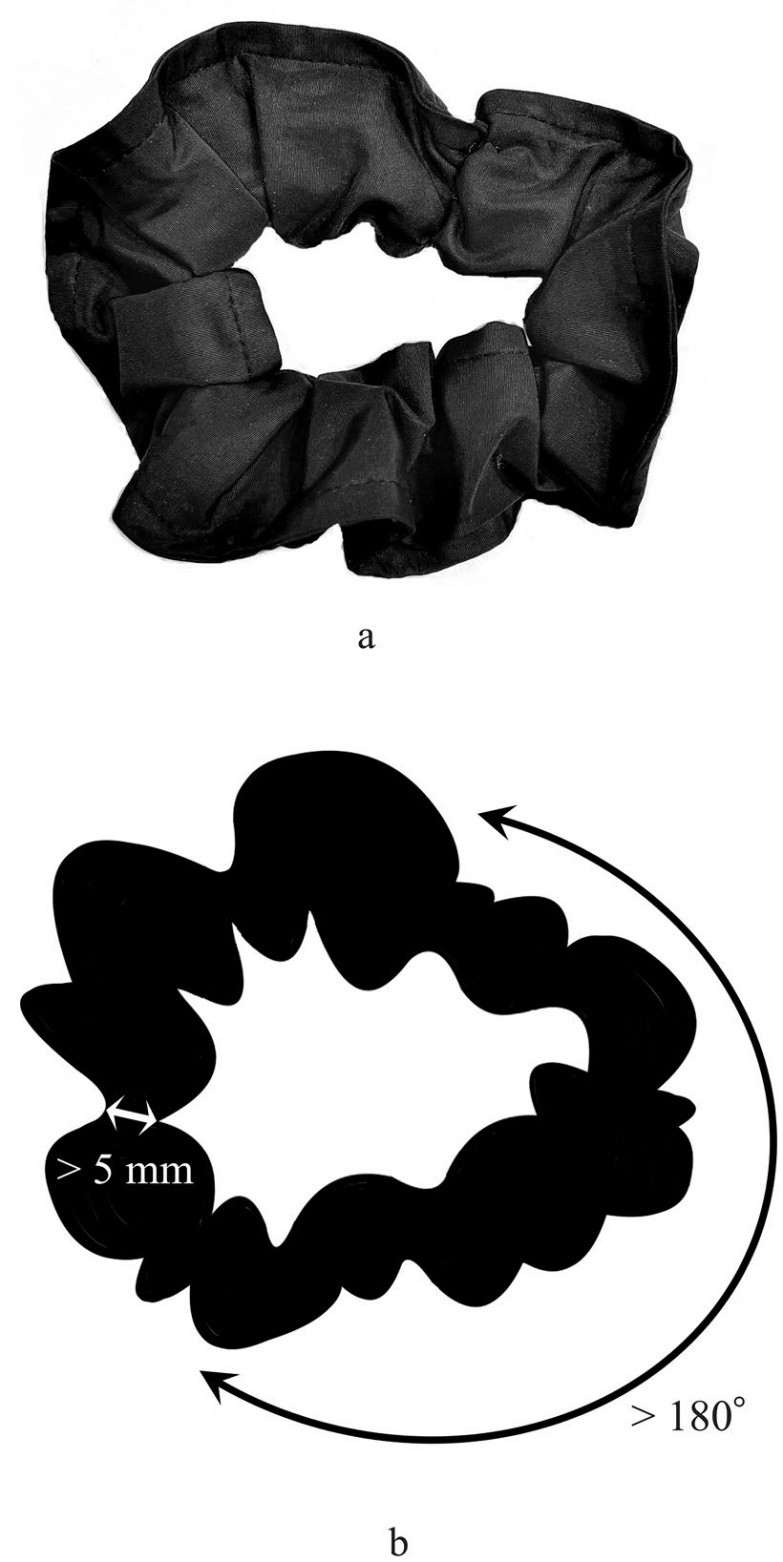
The black scrunchie sign. The black scrunchie sign was defined as a thick (> 5 mm) and lobulated hypointense rim (> 180°) with central hyperintense areas on T2-weighted images. Photo (**a**) and illustration (**b**) of the black scrunchie were exhibited

For qualitative assessments, Radiologists 1 and 2 evaluated the predominance of solid or cystic components, tumor margin (well-defined or ill-defined), and lobulated margin (presence or absence). Predominantly solid lesions were classified as pure solid lesions or solid lesions with cystic components, whereas predominantly cystic lesions were classified as pure cystic lesions or cystic lesions with a mural nodule. Furthermore, we assessed the presence or absence of hypointensity within solid components similar to the skeletal muscle on T2-weighted images and hyperintensity within cystic components relative to the skeletal muscle on T1-weighted images. We evaluated the degree of contrast enhancement compared with the myometrium (mild to moderate or strong) on fat-suppressed contrast-enhanced T1-weighted images. Furthermore, we assessed the presence or absence of black scrunchie or mille-feuille signs, stained-glass appearance, fluid–fluid level, and flow void. The black scrunchie sign was defined as a thick (> 5 mm) and lobulated hypointense rim (> 180°) with central hyperintense areas on T2-weighted images (Fig. 1). If the black scrunchie sign was observed, the reviewers also assessed the presence of the peripheral hyperintense areas on diffusion-weighted images compared with the myometrium, corresponding to hypointense rim on T2-weighted images. The mille-feuille sign was defined as fine layered structures, whose layers were several mm apart, and a width/length of ≥ 10/20 mm [12]. Furthermore, the stained-glass appearance was defined as multilocular cystic masses wherein the loculi revealed variable signal intensity (SI) on T1- and T2-weighted images [13, 14]. Moreover, we assessed the presence or absence of abnormal ascites, peritoneal dissemination, and lymphadenopathy. Abnormal ascites was defined as the presence of ascites exceeding the level of the uterine fundus and/or filling the pelvic cavity, whereas physiological ascites was defined as fluid at the Douglas level [15]. Peritoneal dissemination was defined as nodular or smooth thickening of the peritoneum. Lymph nodes were considered to have lymphadenopathy when a node had a short-axis diameter of more than 8 mm in the pelvis [16].

For quantitative assessments, Radiologist 1 measured all the quantitative parameters. The maximum tumor diameter of the entire lesion was measured using axial, coronal, and sagittal T2-weighted images. If solid and cystic components were observed within the tumor, the maximum diameters of the solid and cystic components were also measured. The SIs of the solid components on T1-, T2-, and fat-suppressed contrast-enhanced T1-weighted images were measured using oval or spherical regions of interest (ROIs), which were manually placed on the solid components as widely as possible in the slice showing the maximum diameter by referring to T2/T1- and/or contrast-enhanced T1-weighted images. In cases without contrast-enhanced images, the reviewer placed ROIs of the solid components, avoiding marked hyperintense areas on T2-weighted images and/or obvious hyperintense areas on T1-weighted images, which indicated fluid collection. The reviewer measured the SI of the skeletal muscles at the same level as that of the lesion by manually placing 20-mm diameter circle ROIs and then the tumor-to-muscle signal intensity ratio (SIR) was calculated. The mean apparent diffusion coefficient (ADC) values of the solid components within the tumor were measured on ADC maps by manually placing oval or spherical ROIs. If the black scrunchie sign was observed, the reviewer also measured ADC values in the central and peripheral areas on ADC maps.

### Histopathological assessment

A pathologist with 12 years of post-training experience microscopically reviewed resected OMSC specimens. The pathologist assessed the predominant histological subtype of stomach cancer according to the 15th edition of the Japanese Gastric Cancer classification. Among OMSCs with the black scrunchie sign, the occupying rates of edematous stroma, fibrous stroma, and tumor cellularity were also assessed in the central and peripheral areas of the tumor.

### Statistical analysis

All statistical analyses were performed using the Statistical Package for the Social Sciences version 24.0 (IBM Corp., Armonk, NY, USA). The Mann–Whitney U test was used to compare the quantitative measurements between OMCCs and OMSCs. Fisher’s exact test was performed to compare the qualitative findings between OMCCs and OMSCs. *p* values < 0.05 were considered significant. Interobserver variability in qualitative assessments was evaluated using Kappa statistic. A Kappa value of ≤ 0.20 was interpreted as slight agreement, 0.21–0.40 as fair agreement, 0.41–0.60 as moderate agreement, 0.61–0.80 as substantial agreement, and ≥ 0.81 as almost perfect agreement.

## Results

### Patients’ characteristics

Table 1 summarizes the patients’ characteristics. No significant difference in patients’ age was observed between the 8 patients with OMSC and 18 patients with OMCC (50.5 ± 12.7 years vs. 56.8 ± 11.8 years, *p* = 0.21). CEA was significantly higher in patients with OMCC than in those with OMSC (250 ± 859 ng/mL vs. 4 ± 5 ng/mL, *p* < 0.01); however, no significant differences were observed in CA125 (*p* = 0.19) and CA19-9 (*p* = 0.18) between patients with OMSC and OMCC. Four of 8 patients (50%) with stomach cancer and 7 of 18 patients (39%) with colorectal cancer had histopathologically proven bilateral ovarian metastasis (*p* = 0.68); therefore, this study enrolled patients with 12 OMSCs and 25 OMCCs. The frequency of abnormal ascites (*p* = 0.31), peritoneal dissemination (*p* = 0.34), and lymphadenopathy (*p* = 0.65) did not significantly differ between patients with OMSC and those with OMCC.

**Table 1:** Patient characteristics

Parameter	Patients with OMSC (*n* = 8)	Patients with OMCC (*n* = 18)	*p* value
Age (year)	50.5 ± 12.7 (34–73)	56.8 ± 11.8 (36–83)	0.21
CEA (ng/mL)	4 ± 5	250 ± 859	0.004*
CA125 (U/mL)	54 ± 52	256 ± 339	0.19
CA19-9 (U/mL)	322 ± 860	1099 ± 3786	0.18
Laterality			
Unilateral	4 (50)	11 (61)	0.68
Bilateral	4 (50)	7 (39)	
Number of OM	12	25	
Abnormal ascites	2 (25)	8 (44)	0.31
Peritoneal dissemination	1 (13)	4 (22)	0.50
lymphadenopathy	0 (0)	1 (6)	0.69

*OMSC* ovarian metastasis from stomach cancer, *OMCC* ovarian metastasis from colorectal cancer, *CEA* serum carcinoembryonic antigen, *CA*125 cancer antigen 125, *CA*19-9 carbohydrate antigen 19–9, *OM* ovarian metastasis

Qualitative data are numbers of patients with percentages in parentheses

Quantitative data are expressed as mean ± standard deviation with ranges in parentheses

^*^Significant differences in the values were observed between OMSC and OMCC (*p* < 0.01)

### MRI findings

**Fig. 2:**
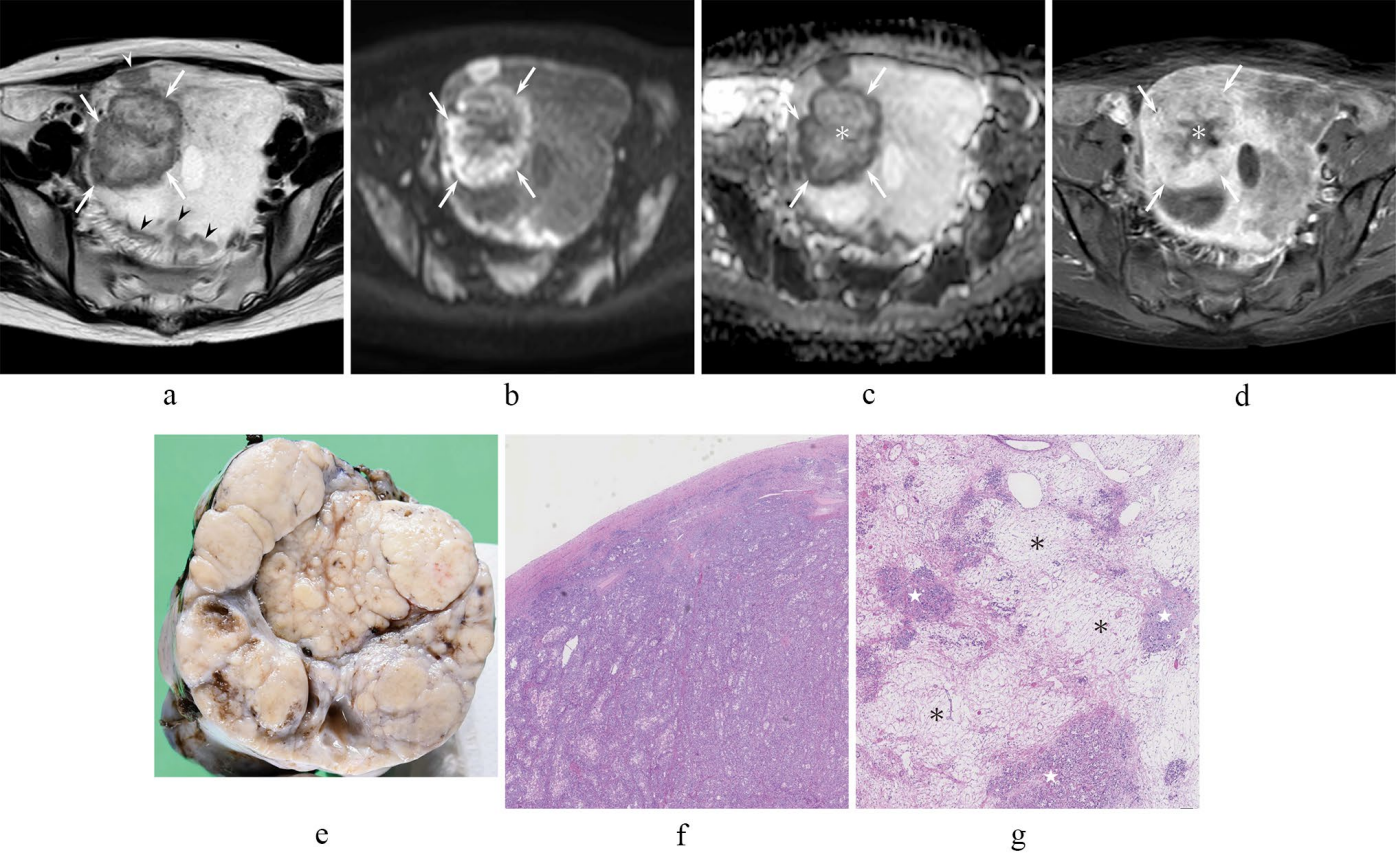
A 34-year-old woman with right OMSC whose predominant histological subtype is por1. **a** T2-weighted image shows a pure solid mass with the black scrunchie sign (arrows). Multiple peritoneal dissemination (arrowheads) and ascites are observed. **b** Diffusion-weighted image shows a solid mass with peripheral hyperintensity (arrows). **c** ADC map shows high ADC value in the central areas (asterisk) and low ADC value in the peripheral areas (arrows). **d** Fat-suppressed contrast-enhanced T1-weighted image shows heterogeneously strong enhancement (arrows) with central unenhanced areas (asterisk). **e** Sectioned gross specimen of right OMSC demonstrates a solid, multilobulated, yellowish-white cut surface. **f** Histological specimen (H&E stain, × 40 magnification) in the peripheral areas of right OMSC shows hypercellular areas. **g** Histological specimen (H&E stain, × 40 magnification) in the central areas of right OMSC shows combined edematous (asterisks) and hypercellular (white stars) areas

**Fig. 3:**
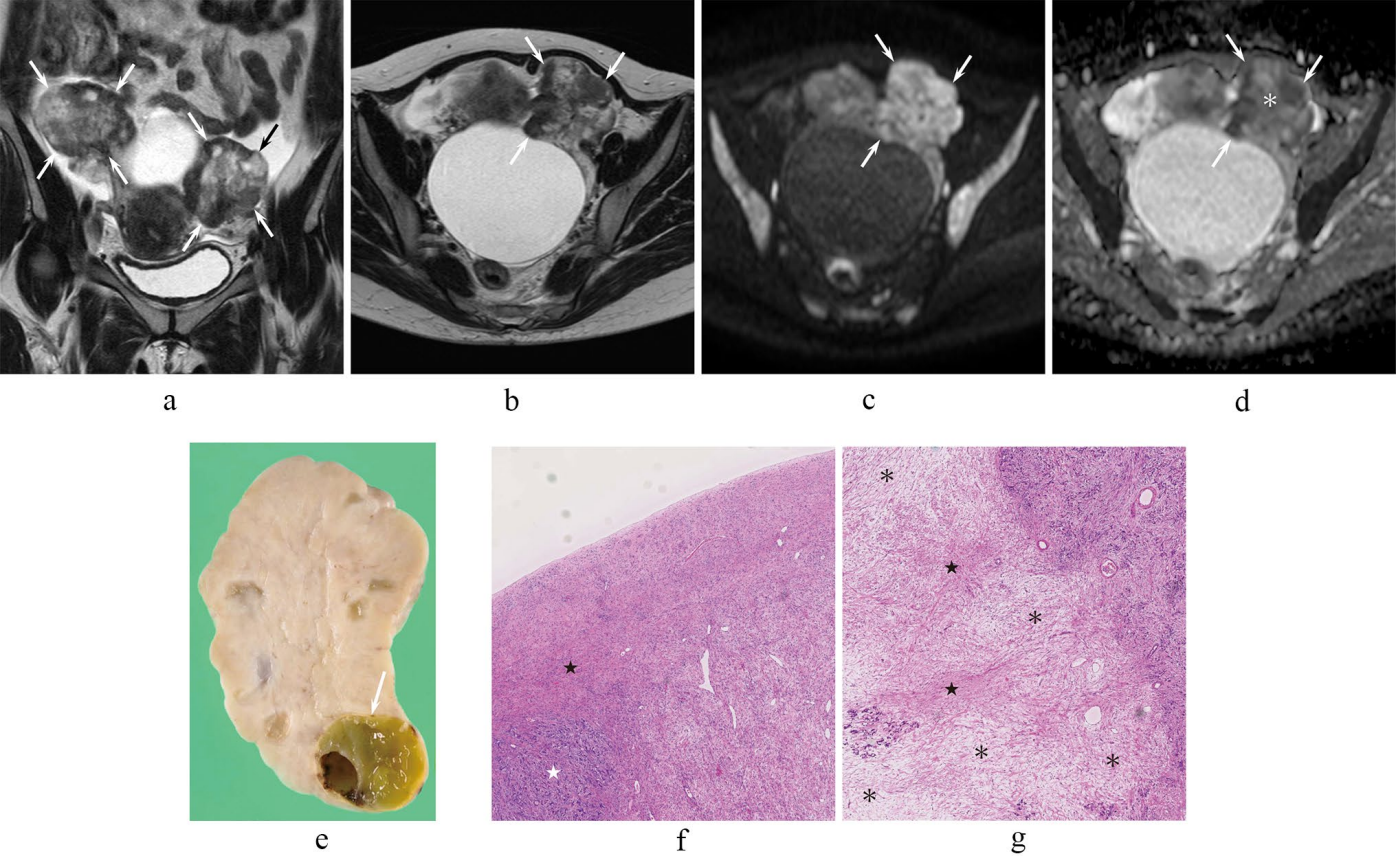
A 47-year-old woman with bilateral OMSCs whose predominant histological subtypes are sig (right) and tub1 (left). **a** Coronal T2-weighted image shows solid masses with small cystic components with black scrunchie sign (arrows). **b** T2-weighted image shows a solid mass of the left ovary with the black scrunchie sign (arrows). **c** Diffusion-weighted image shows a solid mass of the left ovary with peripheral hyperintensity (arrows). **d** ADC map shows high ADC value in the central areas (asterisk) and low ADC value in the peripheral areas (arrows). **e** Sectioned gross specimen of left OMSC demonstrates a solid, yellowish-white cut surface with a cystic area (arrow). **f** Histological specimen (H&E stain, × 40 magnification) in the peripheral areas of left OMSC shows combined fibrous (black star) and hypercellular (white star) areas. **g** Histological specimen (H&E stain, × 40 magnification) in the central areas of left OMSC shows combined edematous (asterisks) and fibrous (black stars) areas

**Fig. 4:**
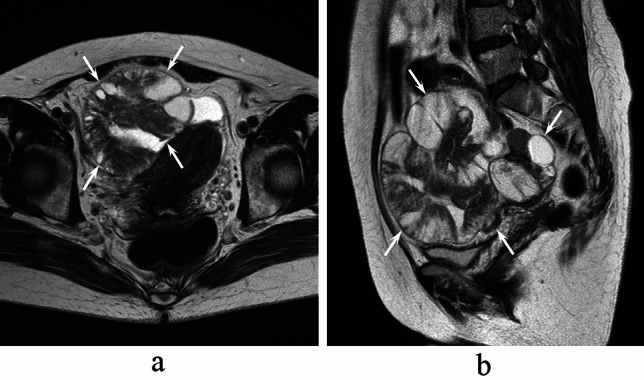
A 50-year-old woman with right OMCC. Axial (**a**) and sagittal (**b**) T2-weighted images show a multilocular cystic mass with the mille-feuille sign (arrows)

**Fig. 5:**
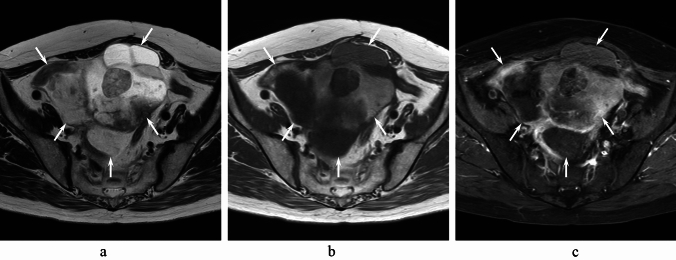
A 69-year-old woman with left OMCC. Axial T2- (**a**), T1- (**b**), and fat-suppressed contrast-enhanced T1- (**c**) weighted images show a multilocular cystic mass with the stained-glass appearance (arrows)

Table 2 summarizes the qualitative results. In all cases, OMSCs (Figs. 2 and 3) were predominantly solid; they could be classified as solid lesions with cystic components (92%) and pure solid lesions (8%). OMCCs (Figs. 4 and 5) were predominantly cystic in 20/25 (80%) and solid in 5/25 (20%) patients, which were classified as cystic lesions with mural nodules (72%), solid lesions with cystic components (16%), pure cystic lesions (8%), and pure solid lesions (4%). Predominantly solid lesions (100% vs. 20%, *p* < 0.01), black scrunchie sign (33% vs. 0%, *p* < 0.01), strong contrast enhancement (80% vs. 10%, *p* < 0.01), and flow void (67% vs. 20%, *p* < 0.01) were significantly more frequently observed in OMSCs than in OMCCs. Among four OMSCs with black scrunchie sign, the peripheral areas corresponding to hypointense rim on T2-weighted images showed hyperintensity on diffusion-weighted images in all four OMSCs (Figs. 2b and 3c) and three OMSCs had flow void. Hyperintense areas within cystic components on T1-weighted images (71% vs. 18%, *p* < 0.01) and stained-glass appearance (44% vs. 0%, *p* < 0.01) were significantly more frequently observed in OMCCs than in OMSCs. However, no significant differences were observed in the tumor margin (*p* = 0.68), lobulated margin (*p* = 0.50), hypointensity within solid components on T2-weighted images (*p* = 0.74), mille-feuille sign (*p* = 0.08), and fluid–fluid level (*p* = 0.25) between OMCCs and OMSCs. The black scrunchie sign (Figs. 2 and 3) was only observed in OMSCs, whereas the mille-feuille sign (Fig. 4) and stained-glass appearance (Fig. 5) were observed in OMCCs alone.

**Table 2:** Qualitative imaging findings of OMSC and OMCC

Parameter	OMSC (*n* = 12)	OMCC (*n* = 25)	*p* value
Predominance			0.000*
Solid	12 (100)	5 (20)	
Cystic	0 (0)	20 (80)	
Configuration			
Pure solid	1 (8)	1 (4)	
Solid with cystic component	11(92)	4 (16)	
Cystic with mural nodules	0 (0)	18 (72)	
Pure cystic	0 (0)	2 (8)	
Well-defined margin	12 (100)	24 (96)	0.68
Lobulated margin	5 (42)	14 (56)	0.50
Hypointensity within solid components on T2WI	6 (50)	13 (56) (*n* = 23)	0.74
Hyperintensity within cystic components on T1WI	2 (18) (*n* = 11)	17 (71) (*n* = 24)	0.005*
Strong contrast enhancement on CET1WI	8 (80) (*n* = 10)	1 (10) (*n* = 10)	0.003*
Black scrunchie sign	4 (33)	0 (0)	0.007*
Mille-feuille sign	0 (0)	6 (24)	0.08
Stained-glass appearance	0 (0)	11 (44)	0.005*
Fluid-fluid level	1 (8)	6 (24)	0.25
Flow void	8 (67)	5 (20)	0.008*

*OMSC* ovarian metastasis from stomach cancer, *OMCC* ovarian metastasis from colorectal cancer, *T*2*WI* T2-weighted images, *T*1*WI* T1-weighted images, *CET*1*WI* contrast-enhanced T1-weighted images

Qualitative data are numbers of patients with percentages in parentheses

^*^Significant differences in the frequencies were observed between OMSC and OMCC (*p* < 0.01)

Table 3 summarizes the quantitative results. SIR of solid components on T2-weighted images (3.30 ± 0.70 vs. 2.52 ± 0.77, *p* < 0.01) and gadolinium-enhanced T1-weighted images (2.21 ± 0.57 vs. 1.43 ± 0.32, *p* < 0.01) were significantly higher in OMSCs than in OMCCs. Although no significant differences were observed in the maximum tumor diameter (93.8 ± 37.5 mm vs. 110.4 ± 47.7 mm, *p* = 0.25) between OMSCs and OMCCs, the maximum diameter of solid components was significantly larger in OMSCs than in OMCCs (80.6 ± 32.0 mm vs. 49.6 ± 23.7 mm, *p* < 0.05) and that of cystic components was significantly larger in OMCCs than in OMSCs (43.6 ± 30.5 mm vs. 86.8 ± 52.2 mm, *p* < 0.05). No significant differences were observed in the SIR of solid components on T1-weighted images (*p* = 0.40) and ADC values (*p* = 0.05). Among four OMSCs with black scrunchie sign, mean ADC value in the central areas was 1.61 × 10^−3^ mm^2^/s (range, 1.49–1.72 × 10^−3^ mm^2^/s), whereas that in the peripheral areas was 1.12 × 10^−3^ mm^2^/s (range, 1.01–1.18 × 10^−3^ mm^2^/s).

**Table 3:** Quantitative imaging findings of OMSC and OMCC

Parameter	OMSC (*n* = 12)	OMCC (*n* = 25)	*p* value
Maximum tumor diameter (mm)	93.8 ± 37.5	110.4 ± 47.7	0.25
Maximum diameter of solid components (mm)	80.6 ± 32.0	49.6 ± 23.7 (*n* = 23)	0.010*
Maximum diameter of cystic components (mm)	43.6 ± 30.5 (*n* = 11)	86.8 ± 52.2 (*n* = 24)	0.012*
SIR of solid components on T2WI	3.30 ± 0.70 (*n* = 12)	2.52 ± 0.77 (*n* = 23)	0.004**
SIR of solid components on T1WI	1.02 ± 0.16 (*n* = 12)	0.96 ± 0.19 (*n* = 23)	0.40
SIR of solid components on CET1WI	2.21 ± 0.57 (*n* = 10)	1.43 ± 0.32 (*n* = 10)	0.001*
ADC value (× 10^–3^ mm^2^/s)	1.52 ± 0.21 (*n* = 11)	1.32 ± 0.35 (*n* = 22)	0.05

*OMSC* ovarian metastasis from stomach cancer, *OMCC* ovarian metastasis from colorectal cancer, *T*2*WI* T2-weighted images, *T*1*WI* T1-weighted images, *CET*1*WI* contrast-enhanced T1-weighted images, *ADC* apparent diffusion coefficient

Quantitative data are expressed as mean ± standard deviation

^*^Significant differences in the values were observed between OMSC and OMCC (*p* < 0.05)

^**^Significant differences in the values were observed between OMSC and OMCC (*p* < 0.01)

The Kappa values for the two reviewers exhibited fair agreement regarding the lobulated margin (0.37) and moderate agreement regarding the tumor margin, hyperintensity within cystic components on T1-weighted images, degree of contrast enhancement, stained-glass appearance, and fluid–fluid level (0.46–0.57). There was substantial or almost perfect agreement was regarding other assessments (0.62–1.00).

### Histopathologic findings

Histopathological assessments are summarized in Table 4. Solid-type poorly differentiated adenocarcinoma (por1, *n* = 4) and non-solid type poorly differentiated adenocarcinoma (por2, *n* = 4) were the predominant histological subtypes of stomach cancer among the 12 OMSCs, followed by well-differentiated tubular adenocarcinoma (tub1, *n* = 3) and signet-ring cell carcinoma (sig, *n* = 1). Among the 4 OMSCs with the black scrunchie sign, the predominant histological subtypes were por1 (*n* = 2), tub1 (*n* = 1), and sig (*n* = 1). A close relationship between the predominant histological subtype of stomach cancer and the black scrunchie sign was not observed.

**Table 4:** Histopathological assessments of OMSC

Tumor no	Age (years)	Black scrunchie sign	Predominant histological subtype of stomach cancer	Central area			Peripheral area		
				Edema	Fibrosis	Cellular	Edema	Fibrosis	Cellular
1	34	+	Por1	40	20	40	10	20	70
2	47	+	Sig	40	40	20	15	75	10
3		+	Tub1	30	65	5	0	90	10
4	61	+	Por1	40	30	30	5	10	85
5	38	−	Por1	NA	NA	NA	NA	NA	NA
6		−	Por1	NA	NA	NA	NA	NA	NA
7	47	−	Por2	NA	NA	NA	NA	NA	NA
8		−	Por2	NA	NA	NA	NA	NA	NA
9	47	−	Por2	NA	NA	NA	NA	NA	NA
10		−	Tub1	NA	NA	NA	NA	NA	NA
11	57	−	Por2	NA	NA	NA	NA	NA	NA
12	73	−	Tub1	NA	NA	NA	NA	NA	NA

*OMSC* ovarian metastasis from stomach cancer, *Por*1 solid-type poorly differentiated adenocarcinoma, *Sig* signet-ring cell carcinoma, *Tub*1 well-differentiated tubular adenocarcinoma, *Por*2 non-solid type poorly differentiated adenocarcinoma, *NA* not available

Numbers in the central and peripheral areas are occupying rates (percentages)

Among the 4 OMSCs with the black scrunchie sign, edematous stroma in the central areas was always more extensive than that in the peripheral areas of the tumor, whereas more prominent fibrous stroma (*n* = 2) or higher tumor cellularity (*n* = 2) were observed in the peripheral areas than in the central areas of the tumor.

## Discussion

Predominantly solid lesions with larger diameters of solid components, black scrunchie sign, flow void, hyperintensity within solid components on T2-weighted images, and strong contrast enhancement were characteristic findings of OMSCs compared with OMCCs. Meanwhile, predominantly cystic lesions with larger diameters of cystic components, hyperintensity within cystic components on T1-weighted images, and stained-glass appearance were characteristics of OMCCs compared with OMSCs. The black scrunchie sign was only observed in OMSCs, whereas the mille-feuille sign and stained-glass appearance were only observed in OMCCs.

In the present study, OMSCs always exhibited predominantly solid lesions, whereas OMCCs usually exhibited predominantly cystic lesions. A previous study evaluating CT findings of ovarian metastasis reported that OMSCs were more solid in nature (cystic, 0% [0/13 lesions]; mainly cystic, 30.8% [4/13 lesions]; mainly solid, 15.4% [2/13 lesions]; solid, 53.8% [7/13 lesions]) than OMCCs (cystic, 6.5% [2/31 lesions]; mainly cystic, 83.9% [26/31 lesions]; mainly solid, 6.5% [2/31 lesions]; solid, 3.2% [1/31 lesions]) [6]. Another study evaluating CT findings of OMCCs reported that had a more cystic nature (cystic: 5.0% [1/21 lesions], mainly cystic: 81.0% [17/21 lesions], mainly solid: 9.5% [2/21 lesions], or solid: 5.0% [1/21 lesions]) [17]. Therefore, OMSCs predominantly exhibited solid as opposed to OMCCs.

A previous study evaluating CT findings of ovarian metastasis reported that the longest diameter in axial CT images of OMCCs (mean, 91 mm) was significantly larger than that of OMSCs (mean, 56 mm) [6]. Other studies reported that the mean longest diameter in axial CT images of OMCCs was 92 mm [17] and the mean tumor size in MRI images was 77 mm [12]. The mean maximum tumor diameter in the present study (94 mm in OMSCs and 110 mm in OMCCs) was larger than that reported in the previous studies. This was because we measured tumor size using axial, coronal, and sagittal T2-weighted images. Although OMCCs tended to be larger than OMSCs, differentiating OMSCs from OMCC based only on tumor size is challenging.

In this study, the black scrunchie sign was occasionally observed in OMSCs but not in OMCCs. Our histological investigation revealed that central hyperintense areas on T2-weighted images corresponded to edematous stroma, whereas peripheral hypointense areas on T2-weighted images corresponded to fibrous stroma or high tumor cellularity. A previous study evaluating MRI findings of Krukenberg tumors reported that 12 of 21 (57%) Krukenberg tumors exhibited various amounts of hypointense solid components in random distribution or predominantly in the periphery on T2- and T1-weighted images, which suggested the presence of induced desmoplastic reaction or fibrosis [7]. Meanwhile, as mentioned below, OMSCs are usually hypervascular; therefore, the central areas of hypervascular OMSCs can become ischemic, thereby decreasing tumor cellularity. The characteristic black scrunchie sign of OMSCs may be caused by these histological features in the central and peripheral tumor sites. However, ovarian metastases from other primary hypervascular tumors, such as breast cancer, renal cell carcinoma, and malignant melanoma, may exhibit the black scrunchie sign. For ovarian metastases to present the black scrunchie sign, fibrous stroma or higher tumor cellularity in the peripheral areas and edematous stroma in the central areas are necessary. Further investigation is required to determine the specificity of the black scrunchie sign in OMSCs.

Takeuchi et al. reported “black garland sign”, which refers to a marked T2 hypointense thick rim of fibrous tissue surrounding the ovary in the setting of ovarian fibromatosis [18]. Ovarian fibromatosis commonly occurs in young women and is characterized by a proliferation of collagen-producing spindle cells surrounding normal ovarian structure; therefore, central ovarian parenchyma is usually accompanied by ovarian follicles. Although the “black garland sign” and “black scrunchie sign” look similar, we proposed “black scrunchie sign” for OMSCs in the present study. One of the characteristic features of OMSCs with “black scrunchie sign” is lobulated margins; however, it's difficult for “black garland sign” to convey the nuances of lobulated margins. Although ovarian fibromatosis with “black garland sign” occasionally lacks lobulated margins [19, 20], “black garland sign” is suitable for ovarian fibromatosis regardless of whether ovarian fibromatosis has lobulated margins because “black garland sign” did not define the marginal configuration. Meanwhile, “black scrunchie sign” must be naming that indicates characteristics of OMSCs, because OMSCs with “black scrunchie sign” always had lobulated margins. In addition, according to only one case report that described diffusion-weighted imaging findings of ovarian fibromatosis, the inner portion of ovarian fibromatosis, which was consistent with normal ovarian stroma, showed hyperintensity, whereas the outer fibrous portion showed hypointensity on diffusion-weighted images [19]. Meanwhile, in the present study, the peripheral areas corresponding to hypointense rim on T2-weighted images showed hyperintensity on diffusion-weighted images and low ADC values, indicating high cellularity. Therefore, SIs in the peripheral areas on diffusion-weighted images may contribute to the differentiation between OMSCs and ovarian fibromatosis.

In the present study, the SIR of solid components on gadolinium-enhanced T1-weighted images and the frequency of strong contrast enhancement and flow voids were significantly higher in OMSCs than in OMCCs. A previous study of contrast-enhanced CT reported that OMSCs displayed more prominent enhancement (none, 0% [0/13 lesions]; mild, 30.8% [4/13 lesions]; moderate, 15.4% [2/13 lesions]; dense, 53.8% [6/13 lesions]) than OMCCs (none, 0% [0/29 lesions]; mild, 6.9% [2/29 lesions]; moderate, 82.8% [24/29 lesions]; dense, 10.3% [3/29 lesions]) [6]. Strong enhancement with initial rapid rise and early washout (type 3 time-intensity curve) on dynamic contrast-enhanced imaging was observed in solid components of Kruckenberg tumors [11]. Stomach cancers are one of representative hypervascular tumors. High expression of vascular endothelial growth factor A (VEGF-A), which is a key contributor in the formation of new blood vessels from preexisting vasculature, was observed in more than half of stomach cancers, and it is intimately relevant to clinicopathological features, including TNM stage, tumor size, positive lymph nodes, and lymphovascular invasion [21]. The hypervascular nature of OMSCs may contribute to the differentiation of OMSCs from other solid ovarian tumors.

In this study, the mille-feuille sign and stained-glass appearance were observed only in OMCCs. A previous study evaluating the differentiation of MRI findings between OMCCs and primary ovarian cancers reported that the mille-feuille sign was more frequently observed in OMCCs than in primary ovarian tumors (8/41 [19.5%] vs. 1/36 [2.8%], *p* = 0.011) [12]. The frequency of the mille-feuille sign in OMCCs in this study (6/25, 24%) was almost consistent with that in the previous study. Therefore, the mille-feuille sign can be considered a low-frequency but high-specificity MRI feature of OMCCs.

Our study had several limitations. First, this was a single-center retrospective analysis, and the number of patients enrolled was relatively small. In particular, the number of histopathologically proven cases among patients with OMSC was considerably small because OMSCs tend to be accompanied by peritoneal dissemination and treated by chemotherapy. This limitation significantly impacts the study's statistical power and generalizability of the findings. Second, the study did not include ovarian metastases from the mucinous histological subtype of stomach cancer. This is an important limitation, as mucinous gastric carcinomas can resemble colon carcinomas, often containing more cystic components. Third, because dynamic contrast-enhanced MRI was performed on six patients only, its usefulness could not be determined. Dynamic contrast-enhanced imaging may reveal the hypervascular nature of OMSCs. Third, the MRI findings were obtained using MRI scanners with different magnetic field strengths (1.5 T or 3 T), which could potentially influence the calculated ADC values.

## Conclusions

Our findings revealed that OMSCs exhibited predominantly solid lesions and had higher SI of solid components on T2- and gadolinium-enhanced T1-weighted images compared with OMCCs. OMCCs presented as cystic lesions, usually accompanied by hyperintense areas within the cystic components on T1-weighted images. The black scrunchie sign was only observed in OMSCs and corresponded to histological features with edematous stroma in the central areas and fibrous stroma or higher tumor cellularity in the peripheral areas of the tumor.

## References

[1] Kubecek O, Laco J, Spacek J, Petera J, Kopecky J, Kubeckova A, et al. The pathogenesis, diagnosis, and management of metastatic tumors to the ovary: a comprehensive review. Clin Exp Metastasis. 2017;34(5):295–307. 28730323 10.1007/s10585-017-9856-8 PMC5561159

[2] Agnes A, Biondi A, Ricci R, Gallotta V, D’Ugo D, Persiani R. Krukenberg tumors: seed, route and soil. Surg Oncol. 2017;26(4):438–45. 29113663 10.1016/j.suronc.2017.09.001

[3] Cho JH, Lim JY, Choi AR, Choi SM, Kim JW, Choi SH, et al. Comparison of surgery plus chemotherapy and palliative chemotherapy alone for advanced gastric cancer with Krukenberg tumor. Cancer Res Treat. 2015;47(4):697–705. 25648093 10.4143/crt.2013.175 PMC4614195

[4] Shi J, Huang A, Song C, Li P, Yang Y, Gao Z, et al. Effect of metastasectomy on the outcome of patients with ovarian metastasis of colorectal cancer: a systematic review and meta-analysis. Eur J Surg Oncol. 2023;49(9): 106961. 37355393 10.1016/j.ejso.2023.06.013

[5] Wu F, Zhao X, Mi B, Feng LU, Yuan NA, Lei F, et al. Clinical characteristics and prognostic analysis of Krukenberg tumor. Mol Clin Oncol. 2015;3(6):1323–8. 26807242 10.3892/mco.2015.634 PMC4665370

[6] Choi HJ, Lee JH, Kang S, Seo SS, Choi JI, Lee S, et al. Contrast-enhanced CT for differentiation of ovarian metastasis from gastrointestinal tract cancer: stomach cancer versus colon cancer. AJR Am J Roentgenol. 2006;187(3):741–5. 16928939 10.2214/AJR.05.0944

[7] Ha HK, Baek SY, Kim SH, Kim HH, Chung EC, Yeon KM. Krukenberg’s tumor of the ovary: MR imaging features. AJR Am J Roentgenol. 1995;164(6):1435–9. 7754887 10.2214/ajr.164.6.7754887

[8] Antila R, Jalkanen J, Heikinheimo O. Comparison of secondary and primary ovarian malignancies reveals differences in their pre- and perioperative characteristics. Gynecol Oncol. 2006;101(1):97–101. 16278010 10.1016/j.ygyno.2005.09.046

[9] Xu Y, Yang J, Zhang Z, Zhang G. MRI for discriminating metastatic ovarian tumors from primary epithelial ovarian cancers. J Ovarian Res. 2015;8:61. 26310488 10.1186/s13048-015-0188-5 PMC4551762

[10] Koyama T, Mikami Y, Saga T, Tamai K, Togashi K. Secondary ovarian tumors: spectrum of CT and MR features with pathologic correlation. Abdom Imaging. 2007;32(6):784–95. 17318680 10.1007/s00261-007-9186-4

[11] Zulfiqar M, Koen J, Nougaret S, Bolan C, VanBuren W, McGettigan M, et al. Krukenberg tumors: update on imaging and clinical features. AJR Am J Roentgenol. 2020;215(4):1020–9. 32755184 10.2214/AJR.19.22184

[12] Kurokawa R, Nakai Y, Gonoi W, Mori H, Tsuruga T, Makise N, et al. Differentiation between ovarian metastasis from colorectal carcinoma and primary ovarian carcinoma: evaluation of tumour markers and “mille-feuille sign” on computed tomography/magnetic resonance imaging. Eur J Radiol. 2020;124: 108823. 31935596 10.1016/j.ejrad.2020.108823

[13] Kaga T, Kato H, Hatano Y, Kawaguchi M, Furui T, Morishige KI, et al. Can MRI features differentiate ovarian mucinous carcinoma from mucinous borderline tumor? Eur J Radiol. 2020;132: 109281. 32961452 10.1016/j.ejrad.2020.109281

[14] Tanaka YO, Nishida M, Kurosaki Y, Itai Y, Tsunoda H, Kubo T. Differential diagnosis of gynaecological “stained glass” tumours on MRI. Br J Radiol. 1999;72(856):414–20. 10474509 10.1259/bjr.72.856.10474509

[15] Hasbay E, Gorgulu G, Sanci M, Ozamrak BG. Role of magnetic resonance imaging in the differentiation of mucinous ovarian carcinoma and mucinous borderline ovarian tumors. Rev Assoc Med Bras (1992). 2023;69(7):e20230110. 37466596 10.1590/1806-9282.20230110 PMC10351997

[16] Mori T, Kato H, Kawaguchi M, Hatano Y, Ishihara T, Noda Y, et al. A comparative analysis of MRI findings in endometrial cancer: differentiation between endometrioid adenocarcinoma, serous carcinoma, and clear cell carcinoma. Eur Radiol. 2022;32(6):4128–36. 35061079 10.1007/s00330-021-08512-6

[17] Choi HJ, Lee JH, Seo SS, Lee S, Kim SK, Kim JY, et al. Computed tomography findings of ovarian metastases from colon cancer: comparison with primary malignant ovarian tumors. J Comput Assist Tomogr. 2005;29(1):69–73. 15665686 10.1097/01.rct.0000149958.86165.ca

[18] Takeuchi M, Matsuzaki K, Sano N, Furumoto H, Nishitani H. Ovarian fibromatosis: magnetic resonance imaging findings with pathologic correlation. J Comput Assist Tomogr. 2008;32(5):776–7. 18830110 10.1097/RCT.0b013e318157689a

[19] Takeshita N, Tada T, Tokunaga M, Okimura A, Makihara M, Takeshita T. Bilateral ovarian fibromatosis diagnosed with magnetic resonance imaging: a case report. Osaka City Med J. 2019;65(2):135–40.

[20] Montoriol PF, Bayol B. Ovarian fibromatosis: the “black garland” sign. Diagn Interv Imaging. 2020;101(4):259–60. 31711935 10.1016/j.diii.2019.10.004

[21] Wei B, Tai Y, Tong H, Wen SL, Tang SH, Huan H, et al. Correlations between VEGF-a expression and prognosis in patients with gastric adenocarcinoma. Int J Clin Exp Pathol. 2017;10(8):8461–9. 31966698 PMC6965380

